# Supplementary material for: Delivery and Transcriptome Assessment of an In Vitro Three-Dimensional Proximal Tubule Model Established by Human Kidney 2 Cells in Clinical Gelatin Sponges
Source: Int J Mol Sci. 2023 Oct 24;24(21):15547. doi: 10.3390/ijms242115547 (PMC10650118; doi:10.3390/ijms242115547)
Supplement: Supplementary file 1 [file ijms-24-15547-s001.zip › ijms-2650392-supplementary.pptx]

## Slide 1
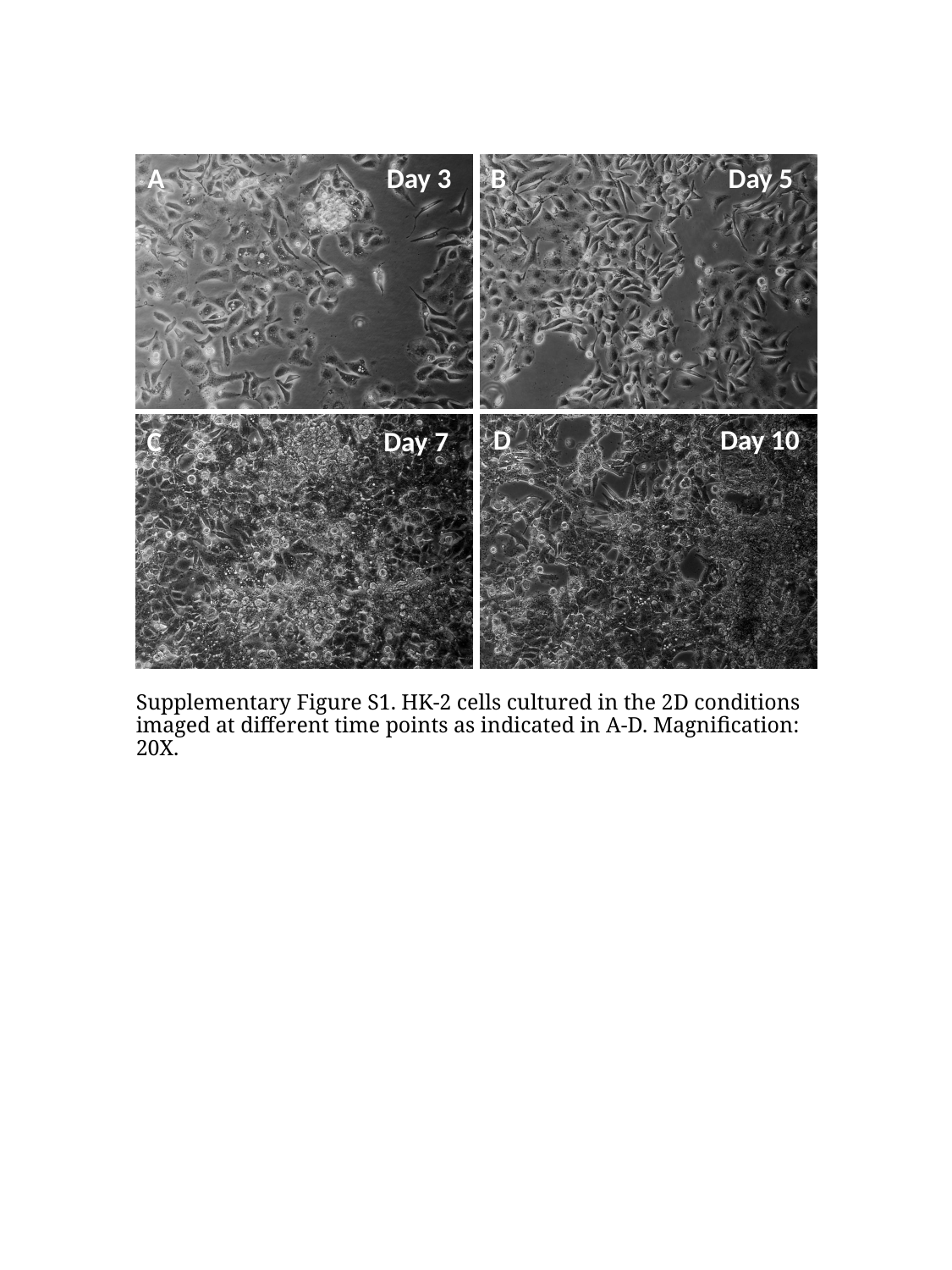

A Day 3
B Day 5
D Day 10
C Day 7
# Supplementary Figure S1. HK-2 cells cultured in the 2D conditions imaged at different time points as indicated in A-D. Magnification: 20X.

## Slide 2
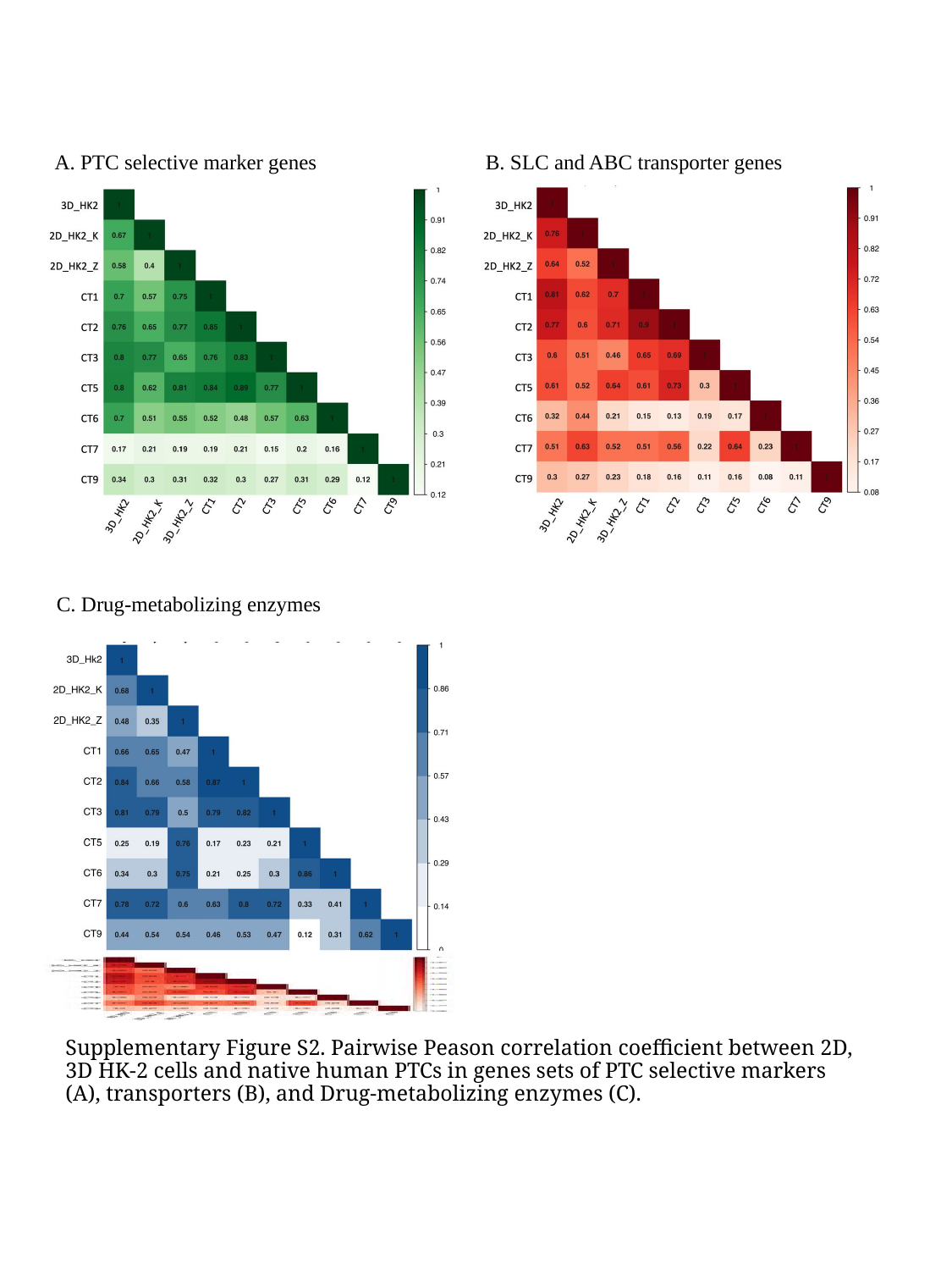

A. PTC selective marker genes
B. SLC and ABC transporter genes
C. Drug-metabolizing enzymes
# Supplementary Figure S2. Pairwise Peason correlation coefficient between 2D, 3D HK-2 cells and native human PTCs in genes sets of PTC selective markers (A), transporters (B), and Drug-metabolizing enzymes (C).

## Slide 3
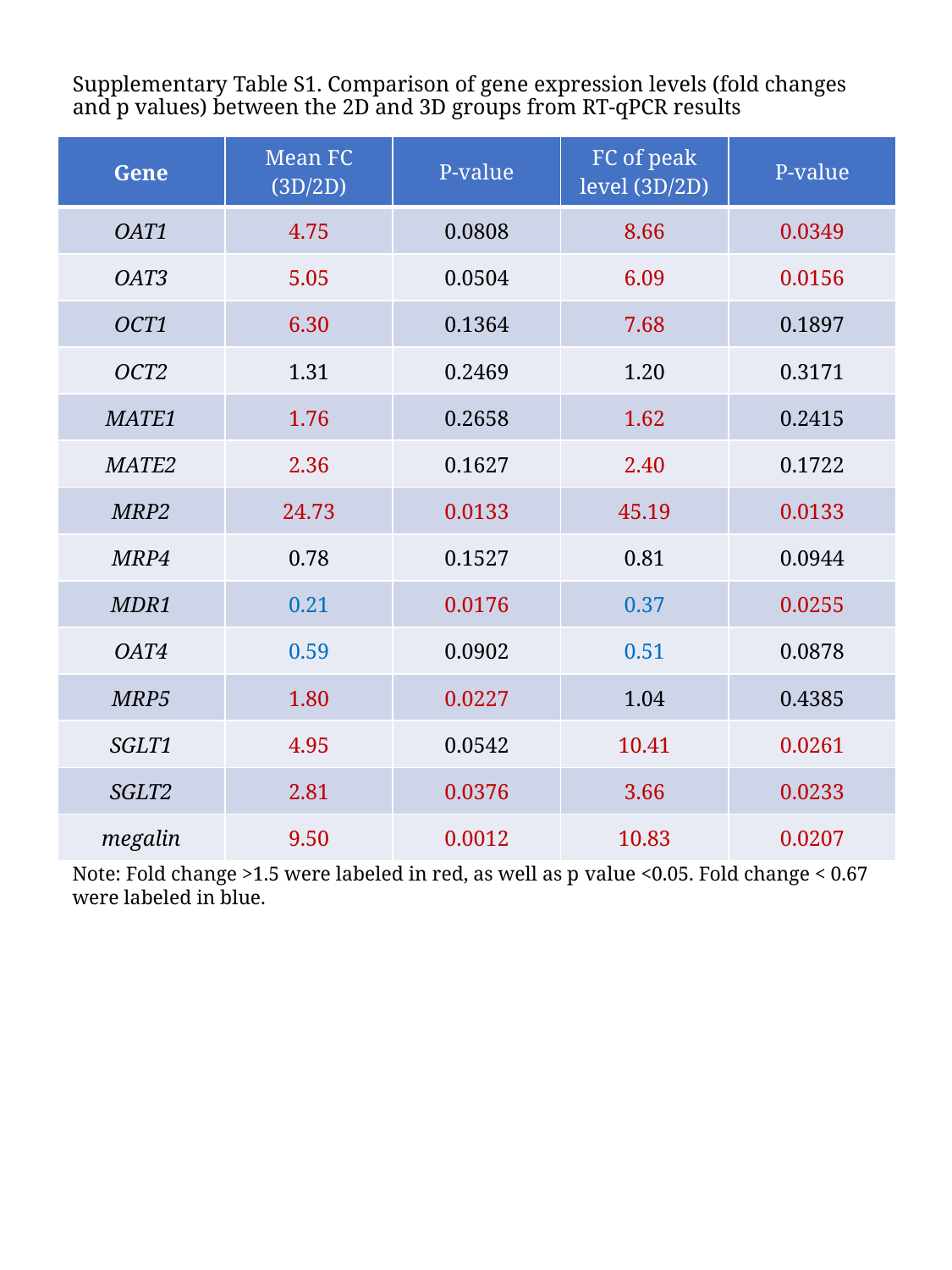

# Supplementary Table S1. Comparison of gene expression levels (fold changes and p values) between the 2D and 3D groups from RT-qPCR results
| Gene | Mean FC (3D/2D) | P-value | FC of peak level (3D/2D) | P-value |
| --- | --- | --- | --- | --- |
| OAT1 | 4.75 | 0.0808 | 8.66 | 0.0349 |
| OAT3 | 5.05 | 0.0504 | 6.09 | 0.0156 |
| OCT1 | 6.30 | 0.1364 | 7.68 | 0.1897 |
| OCT2 | 1.31 | 0.2469 | 1.20 | 0.3171 |
| MATE1 | 1.76 | 0.2658 | 1.62 | 0.2415 |
| MATE2 | 2.36 | 0.1627 | 2.40 | 0.1722 |
| MRP2 | 24.73 | 0.0133 | 45.19 | 0.0133 |
| MRP4 | 0.78 | 0.1527 | 0.81 | 0.0944 |
| MDR1 | 0.21 | 0.0176 | 0.37 | 0.0255 |
| OAT4 | 0.59 | 0.0902 | 0.51 | 0.0878 |
| MRP5 | 1.80 | 0.0227 | 1.04 | 0.4385 |
| SGLT1 | 4.95 | 0.0542 | 10.41 | 0.0261 |
| SGLT2 | 2.81 | 0.0376 | 3.66 | 0.0233 |
| megalin | 9.50 | 0.0012 | 10.83 | 0.0207 |
Note: Fold change >1.5 were labeled in red, as well as p value <0.05. Fold change < 0.67 were labeled in blue.
